# Failure of Miltefosine Treatment for Visceral Leishmaniasis in Children and Men in South-East Asia

**DOI:** 10.1371/journal.pone.0100220

**Published:** 2014-06-18

**Authors:** Bart Ostyn, Epco Hasker, Thomas P. C. Dorlo, Suman Rijal, Shyam Sundar, Jean-Claude Dujardin, Marleen Boelaert

**Affiliations:** 1 Department of Public Health, Institute of Tropical Medicine, Antwerp, Belgium; 2 Division of Pharmaco-epidemiology and Clinical Pharmacology, Utrecht University, Utrecht, The Netherlands; 3 Department of Internal Medicine, B. P. Koirala Institute of Health Sciences, Dharan, Nepal; 4 Department of Medicine, Institute of Medical Sciences, Banaras Hindu University, Varanasi, India; 5 Department of Biomedical Sciences, Institute of Tropical Medicine, Antwerp, Belgium; Singapore Immunology Network, Agency for Science, Technology and Research (A*STAR), Singapore

## Abstract

**Background:**

High frequency of relapse in miltefosine-treated visceral leishmaniasis (VL) patients in India and Nepal followed up for twelve months.

**Objective:**

To identify epidemiological and clinical risk factors for relapse of VL in patients recently treated with standard dosing of miltefosine in India and Nepal.

**Design:**

Prospective observational study in three Primary Health Centers and one reference center in Muzaffarpur district, Bihar, India; and two zonal hospitals and a university hospital in South-east Nepal; records of all consenting patients diagnosed with VL and treated with miltefosine according to the current treatment guidelines of the Kala azar elimination program between 2009 and 2011.

**Results:**

We compared the clinical records of 78 cases of relapse with those of 775 patients who had no record of subsequent relapse. Relapse was 2 times more common amongst male patients (IRR 2.14, 95% CI 1.27–3.61), and 2 to 3 times more frequent in the age groups below 15 compared to the over 25 year olds (age 10 to 14: IRR 2.53; 95% CI 1.37–4.65 and Age 2 to 9: IRR 3.19; 95% CI 1.77–5.77). History of earlier VL episodes, or specific clinical features at time of diagnosis such as duration of symptoms or spleen size were no predictors of relapse.

**Conclusions:**

Young age and male gender were associated with increased risk of VL relapse after miltefosine, suggesting that the mechanism of relapse is mainly host-related i.e. immunological factors and/or drug exposure (pharmacokinetics). The observed decrease in efficacy of miltefosine may be explained by the inclusion of younger patients compared to the earlier clinical trials, rather than by a decreased susceptibility of the parasite to miltefosine. Our findings highlight the importance of proper clinical trials in children, including pharmacokinetics, to determine the safety, efficacy, drug exposure and therapeutic response of new drugs in this age group.

## Introduction

Visceral leishmaniasis (VL) is a systemic parasitic disease that is caused by the *Leishmania donovani* species complex and is typically fatal unless treated. With effective drug treatment, clinical cure is relatively rapidly obtained, suppressing (but not eliminating) the parasite load to undetectable levels, and resulting in a life-long cellular Th1-dependent immune response [1]. However, some VL patients develop post-kala azar dermal leishmaniasis (PKDL) years after being successfully treated, and others relapse with clinical VL, usually within months after the end of treatment. This relapse is common in HIV co-infected patients but also occurs in immunocompetent individuals.

Miltefosine has been recommended as the first-line drug for treatment of leishmaniasis in the VL-endemic regions of India, Nepal and Bangladesh, because of its ease of use and the possibility for ambulatory care, and is now widely used [2]. But from the start, due to its long elimination half-life and the risk of non-compliance under non-observed ambulatory treatment and frequent (mainly gastro-intestinal) side effects, there was also an apprehension of possible emergence of resistance [3]. Close monitoring of the treatment performance under programme conditions would therefore be appropriate, which implies verifying if patients are effectively cured. In practice this requires a clinical check-up of all treated VL patients at (various) time point(s) after the end of their treatment, since cure in VL is a clinical concept, based on remission of the symptoms and absence of relapse in the months following treatment, but there is no laboratory test of cure. We developed and piloted a tool kit for registering early and late treatment outcomes of VL at point-of-care level [4].

In the VL elimination programme in this region there is little attention to the monitoring of the clinical outcomes be it immediately at the end of treatment, or even more so in the months following [5]. Yet, long-term follow-up of patients has proven feasible for other diseases such as tuberculosis and HIV/AIDS care and is also required for non-infectious diseases such as hypertension and diabetes [6], [7].

We recently reported high relapse rates in HIV-negative VL patients treated with miltefosine in non-supervised, ambulatory treatment in Nepal [8]. In this cohort of 120 VL patients, enrolled at a third-line university hospital, 24 patients relapsed within 12 months after completion of treatment (20%, 95% C.I. 12.8–27.2). No significant risk factors were found apart from having an age below 12 years (IRR = 2.43, 95% C.I. 1.09–5.42). Also in India, in a more controlled setting where 567 VL patients were treated with miltefosine under directly observed treatment (DOT), a substantial increase in the failure rate was noted compared to the phase III trial that led to regulatory approval of the drug in India more than a decade ago [9]. True miltefosine-resistant strains could not be identified in any of the clinical parasite isolates obtained from relapsed patients [8], [10]. To get a better understanding of the host determinants of this increased failure rate of VL patients following miltefosine treatment on the Indian subcontinent, we evaluated clinical and epidemiological risk factors for treatment failure in a larger cohort of patients treated with miltefosine, in the framework of the Kaladrug-R study (New Tools for monitoring drug resistance and treatment response in Visceral Leishmaniasis in the Indian subcontinent).

## Materials and Methods

### Ethics Statement

Written informed consent on the use of the anonymized epidemiological, clinical and prospective clinical data was obtained from each patient or their guardian for those aged under 18. Clearance was obtained from the ethical committees of Institute of Medical Sciences (Banaras Hindu University), B P Koirala Institute of Health Sciences, the Institute of Tropical Medicine, and the University of Antwerp.

We followed VL patients prospectively and documented early and late treatment outcome as described in detail in Ostyn et al. (2013) [4]. In brief, patients were enrolled between 2009 and 2011 in seven different centers: three Primary Health Centres (PHCs) in Muzaffarpur district, Bihar India, in two district hospitals in Nepal, and two reference centers: KAMRC in Muzaffarpur, and BPKIHS in Dharan, Ghopa, Nepal. All patients were treated free of cost according to standard national guidelines, with the same proprietary drug (Impavido, Paladin Labs Inc., Montreal, Canada, 50-mg and 10-mg capsules) with the following dosing regimen: 100 mg daily (one 50-mg capsule in the morning and one 50-mg capsule in the evening after meals), for patients weighing >25 kg; 50 mg every morning, for patients weighing ≤25 kg; and 2.5 mg/kg daily in divided doses, for patients aged <12 years. We obtained data on potential clinical and epidemiological risk factors for all patients on a specially designed case record form. Only for the patients treated at the tertiary care centers KAMRC and BPKIHS, more extensive clinical data were available. Clinical endpoints were documented at the end of treatment and at 6 months post-treatment at the PHCs and the district hospitals, and up to 12 months post-treatment at the reference centers KAMRC and BPKIHS, according to the case definitions given in table 1.

**Table 1:** Case definitions for the treatment outcome recording of VL patients.

Early treatment outcomes	
* Initial cure:*	Treatment completed, clinical improvement (absence of fever, regression of enlarged spleen+return of appetite and/or gain in body weight).
* Non-response:*	Signs and symptoms of VL persist or recur+confirmation by a positive smear.
* Defaulter:*	VL case who did not complete the 28 day treatment regimen of Miltefosine and/or did not present for assessment after treatment in the facility where they were enrolled.
* Side-effects related switch:*	Side effects requiring Miltefosine stop and change of treatment.
* Death:*	Any death, whether or not related to KA
**Late treatment outcomes**	
* Definite cure*:	VL case with initial cure and no clinical signs (fever, or increase in spleen size since last visit), six/twelve months after completion of therapy
* Relapse*:	VL case with initial cure but with reappearance of clinical symptoms and/or signs along with smear positive for LD bodies during the six/twelve months of follow up
* Lost to follow-up:*	VL patient who completed therapy but who did not present/could not be traced for assessment at six/twelve months post-treatment.
* Death:*	Any death, whether or not related to KA

**Note:** Treatment Failure: includes both non-response and relapse.

Adapted from TDR/WHO. Indicators for monitoring and evaluation of the kala-azar elimination programme. 2010.

We calculated the cure and failure rates at the end of treatment and at 6 and 12 months post-treatment in an intent-to-treat (ITT) and per-protocol (PP) perspective (definitions for ITT and PP analysis are provided in Table S1). For the per-protocol analysis, only patients with a complete 28 days treatment were considered. For the 6 and 12 months post-treatment outcomes, we included only those patients with a complete follow-up in the PP analysis, while in ITT, all lost-to-follow ups were considered as failures.

Data were analyzed in Stata/IC V10.1 (Stata Corp., College Station Tx, USA). A mixed effects Cox regression model with ‘facility’ as random effect was fitted to test for associations between potential risk factors and relapse. Patients were censored at the time of their last follow-up visit, which could be at 6 months or at 12 months. All variables significant at p = 0.10 level on bivariate analysis were tested in the multivariate model; only factors significant a p = 0.05 level were retained. We tested for interactions among the factors retained in the final model. Kaplan Meyer survival graphs were fitted for the factors retained in the final model. Patients who did not complete the full treatment (i.e. because of default, treatment switch because of severe adverse events, transfer to another health structure and death) were excluded from this part of the analysis, as well as those for whom the late treatment outcome was missing (i.e. lost to follow-up). Characteristics of included and excluded patients are shown in Table S2).

## Results

A total of 1016 patients were treated with miltefosine in the seven health structures within the study period 2009–2011 (table 2). ITT analysis at end of treatment gave a cure rate of 94.0% (95% CI 92.5%–95.5%) (see table 3). Cure rate at 6 months post treatment was 86.4% in ITT (95% CI 84.2%–88.7%) and 93.4% in PP (95% CI 91.8%–95.1%). The ITT worst case analysis scenario resulted in a cure rate of 78.4% (95% CI 75.9%–81.0%). Relapse rate was 6.2% in per protocol analysis and 14.3% in ITT worst-case-scenario (i.e. if all lost to follow-up were failures). There was a high inter-clinic variability in the completeness of the late outcome data, with a very high level of loss-to-follow-up in the district hospitals in Nepal situated close to the border with India (see Figure S1 for details). This was due to a large proportion of the patients treated there being Indian, who returned to India immediately after, or in some cases already during treatment. This rendered correct follow-up and tracing in case of non-attendance to appointments impossible. The fact that no cases of relapse were recorded from these health facilities is obviously biased for that reason.

**Table 2:** Number of cases and completeness of follow-up per health facility.

Health facility	Totaltreated	Treatment notcompleted* (%)	6 M treatmentoutcome unknown	12 M treatmentoutcome unknown
Total	1016	70 (6.9%)	90 (8.9%)	
Kanti PHC (India)	76	9	4 (5.3%)	(not done)
Kudhani PHC (India)	63	6	3 (4.8%)	(n.d.)
Motipur PHC (India)	107	20	1 (0.9%)	(n.d.)
Jaleshwor Distr. Hosp (Nepal)	115	16	63 (54.8%)	(n.d.)
Siraha Distr. Hosp. Lahan (N)	36	1	16 (44.4%)	(n.d.)
KAMRC Muzaffarpur (India)	468	9	0	80 (17.1%)
BPKIHS Dharan (Nepal)	151	9	3 (2.0%)	10 (6.6%)

* = Treatment not completed = defaulter, transfer out, death during treatment, adverse event-related switch.

**Table 3:** Cure rates and relapse rates at various time points under ITT, ITT worst case, and per protocol analysis.

	no cure	cure	total	% cured	95% C.I.	% relapsed	95% C.I.
**End of Treatment**							
ITT	61	955	1016	94,0%	92.54%–95.46%	Na	
PP	4	942	946	99,6%	99.16%–99.99%	Na	
**6 months post-treatment**							
ITT	125	797	922	86,4%	84.23%–88.65%	6,0%	4.44%–7.49%
ITT worst-case	219	797	1016	78,4%	75.92%–80.97%	14.3%	12.12%–16.42%
PP	56	796	852	93,4%	91.76%–95.09%	6,2%	4.60%–7.84%
**12 months post-treatment**							
ITT	80	440	520	84,1%	80.99%–87.27%	12,8%	9.94%–15.68%
ITT worst-case	93	440	533	82,1%	78.83%–85.34%	14,2%	11.22%–17.14%
PP	65	439	504	86,6%	83.62%–89.57%	12,8%	9.90%–15.74%

For the Cox regression analysis we compared the clinical characteristics of the 78 relapse cases with the 775 patients who were considered cured at the last follow-up visit (either at 6 or 12 months post-treatment). (Note that the ratio *relapsed* to *cured* may not represent reality, since 10% of treated patients were lost-to-follow-up, and the maximal follow-up time of those who were cured was not equal between settings).

Relapse was 2 times more common amongst men compared to women, and 2 to 3 times more frequent in the age groups below 15 compared to the over 25 year olds, in the bivariate model (Table 4) as well as in the multivariate analysis (table 5). We tested for interaction between the variables retained and there was none. Previous VL history, or clinical characteristics at time of diagnosis such as duration of symptoms or spleen size were no predictors of relapse.

**Table 4:** Factors associated with relapse in a ‘bi-variate’ model (controlled for treatment facility).

Factors	Cured (n = 775)	Relapsed (%) (n = 78)	IRR	95% CI	*P*-value
Gender					
Female	309	19 (5.8)	Referent		
Male	466	59 (11.2)	1.95	1.16–3.28	0.012
Age group					
25 years and older	348	21 (5.7)	Referent		
15 to 24	143	10 (6.5)	1.12	0.52–2.39	0.775
10 to 14	155	22 (12.4)	2.36	1.29–4.35	0.006
2 to 9	129	25 (16.2)	3.10	1.71–5.59	0.000
Previous Treatment for KA					
No	670	65 (8.8)	Referent		
Yes	93	10 (9.7)	1.09	0.57–2.06	0.793
Duration of symptoms					
8 weeks or less	617	62 (9.1)	Referent		
More than 8 weeks	143	13 (8.3)	0.61	0.33–1.13	0.116
Spleen size at admission >4 cm					
4 cm or less	445	48 (9.7)	Referent		
5 cm or more	288	30 (9.4)	0.80	0.40–1.59	0.524
Missing	42	0			
Reporting of side effects during treatment					
No	617	67 (9.8)	Referent		
Yes	158	11 (6.5)	0.65	0.31–1.33	0.234
Use of pediatric tablets in age ≤12					
No	90	16 (15.1)	Referent		
Yes	89	19 (17.6)	1.35	0.66–2.76	0.409

**Table 5:** Factors associated with relapse in a multivariate model.

Factors	IRR	95% CI	*P*-value
Male sex	2.14	1.27–3.61	0.004
Age 15 to 24	1.06	0.49–2.26	0.883
Age 10 to 14	2.53	1.37–4.65	0.003
Age 2 to 9	3.19	1.77–5.77	<0.0005

**Figure 1:**
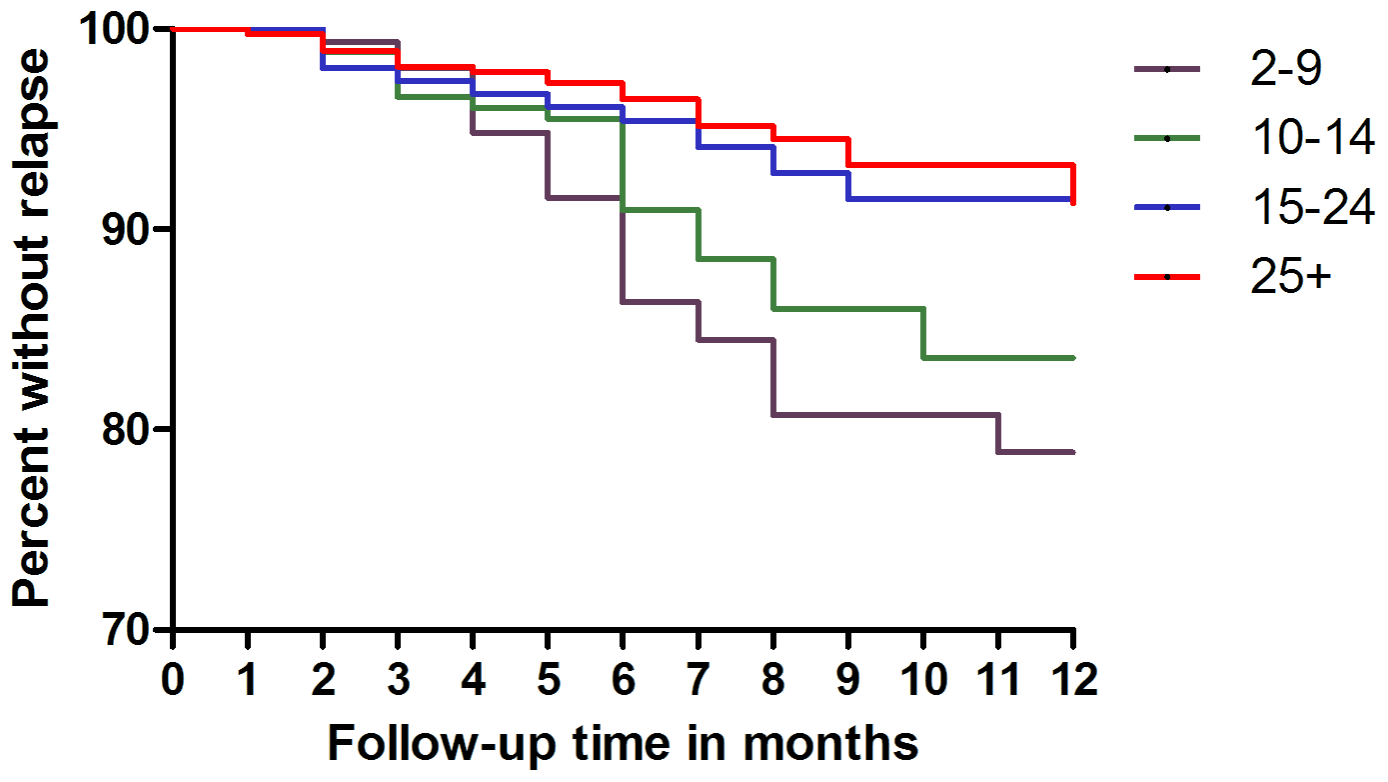
Kaplan-Meier Survival plot for relapse per age group.

**Figure 2:**
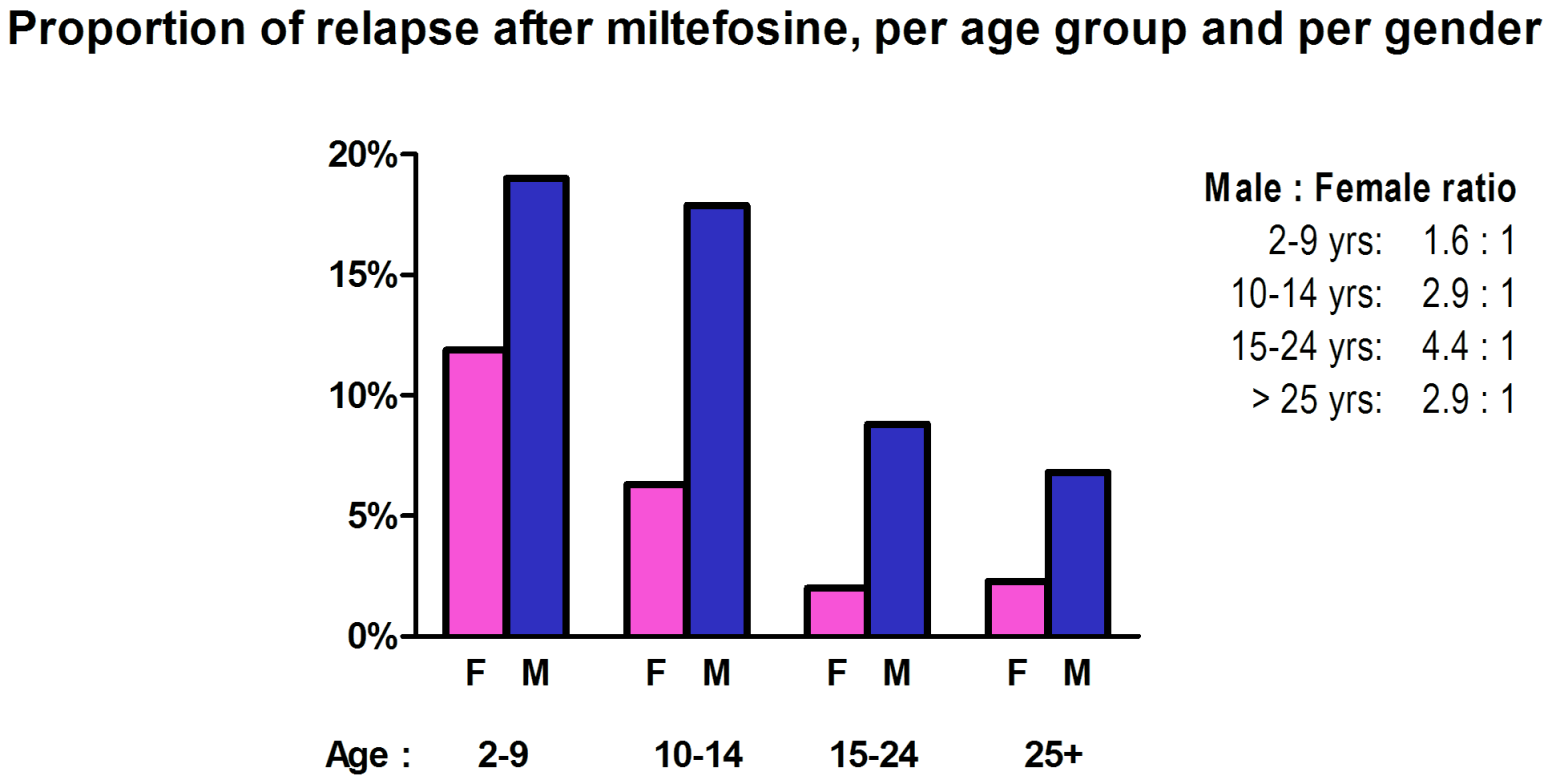
Percentage of Relapse per age and gender.

The Kaplan-Meier survival analysis (figure 1) shows how relapse is more common with younger age, but time of relapse (early vs late) does not differ, and continues to occur after the classic six months’ follow up in all of the age groups. In all age groups, relapse is more common among the males (figure 2).

## Discussion

Our analysis of a cohort of 1016 patients treated with miltefosine in different settings in Bihar, India and neighboring Nepal shows a high relapse rate of 6.2% and 12.8% in PP analysis at 6 and 12 months post-treatment respectively. Clinical and epidemiological risk factors for relapse, considering only those with a post-treatment follow-up time of at least 6 months, were younger age and male sex. Relapse was 2 times more common amongst men (IRR 2.14, 95% CI 1.27–3.61), and 2 to 3 times more frequent in the age groups below 15 years compared to the over 25 year olds (Age 10 to 14: IRR 2.53; 95% CI 1.37–4.65 and Age 2 to 9: IRR 3.19; 95% CI 1.77–5.77). Previous VL history, or clinical presentation at time of diagnosis such as duration of symptoms or spleen size were no predictors of relapse.

Leishmaniasis relapse is known to occur frequently in HIV co-infected patients or in other immunocompromised patients, and when it occurs in immunocompetent patients, it is attributed mainly to drug resistance or suboptimal treatment regimens, as seen with antimonials in Bihar, India [11] and in Southern Sudan [12]. In our study area HIV co-infection is rare, HIV is tested for and when positive, patients are treated with amphotericin B, so our results on miltefosine-treatment concern an HIV-negative population. No arguments for resistance to miltefosine were found [8], [12], [13] and quality, dosing and duration as well as adherence to miltefosine treatment were verified [4], [8], [10], [14] In absence of these explanations, the mechanism of relapse is thought to relate to a failure of maintaining the initially successfully acquired T-cell dependent immune response. In mouse experiments, Murray *et al.* found distinguishable host mechanisms at T cell, cytokine and macrophage levels between the initial and the post-treatment response [15]. *L. donovani* parasites are capable of manipulating the host’s T-cell dependent immune response and in this context, strains with higher infectivity have been have been observed in relapse patients compared to those who did not relapse [16]. Ultimately, many epidemiological and clinical factors that influence immunocompetence might present as risk factors for initial development of VL as well as for treatment failure and eventual relapse of disease, such as age, sex, malnutrition or concomitant infections.

Similarly, age, sex, nutrition and concomitant medication might theoretically have an impact on the metabolism and disposition of miltefosine and reduce exposure to miltefosine [17].

Age and sex have often been cited in epidemiological studies as risk factors for VL as well as for relapse, both in East-African and in Indian VL [18], [19]. Then again in the largest retrospective study of risk factors for VL relapse, Gorski *et al*. in Southern Sudan found, apart from shorter treatment duration, only splenomegaly to be associated with increased risk of relapse, and no association with sex, age, malnutrition and complications of treatment [12]. Note that VL in East-Africa occurs at a younger age than in South-East Asia and can therefore not be compared. Comparable risk factor studies from South-East Asia are not available.

If younger age is indeed a risk factor for relapse, then this could partly explain the increase of the failure rate after oral miltefosine treatment, compared to the phase III trial that had led to the registration of the drug a decade ago [10], since inclusion criteria for age have been changed since. Miltefosine had shown excellent efficacy in the region, notably in clinical trial settings (adults, under DOT, follow-up time of 6 months) with cure rates of over 90% and relapse rates below 5% [20], [21] (see supplementary data, tables 6 and 7). The pediatric studies, though low in number of patients treated, showed poorer outcomes and higher relapse rates from the start [22], and a subsequent report on miltefosine efficacy that included children below 12 years of age, representing 38% of the treated population, also showed poorer outcomes in children [21].

**Table 6:** Overview of various clinical trials on miltefosine efficacy.

Study	Country	Year	Study type	No.	% below 12	Early treatment Outcome			Late treatment Outcome*		
						Clinicalcure (%)	failure/death	missing/ttmswitch	Cure (%)	Relapse (%)	Lost to Follow-up/i.death#
Jha *et al.* (1999) [38]	India	<1999	Phase 2	30	**0**	30 (100%)	0/0	0/0	29 (96.7%)	1 (3.3%)	0/0
Sundar *et al.* (2002) [20]	India	1999–2000	Phase 3	299	**0**	299 (100%)	0/0	0/0	282 (94.3%)	9 (3.0%)	8/0
Bhattacharya *et* *al.* (2007) [21]	India	2003–2004	Phase 4	1132	**38%**	1078 (95.2%)	6/3	45/0	927 (81.9%)	44 (3.9%)	107/0
Rahman *et al.* (2011) [39]	Bangladesh	2006–2007	Phase 4	977	**41%**	865 (88.5%)°	24/0	52/36	701 (71.7%)°	NA	69/0
Sundar *et al.* (2012) [9]	India	2009–2011	Phase 4	567	**24%**	553 (97.5%)	2	4/8	512 (90.3%)	39 (6.9%)	0/2
Rijal *et al.* (2013) [8]	Nepal	2008–2011	Phase 4	120	**28%**	115 (95.8%)	0/1	1/3	99 (82.5%)	13 (10.8%)	0/2
After 12 months:									88 (73.3%)	24 (20.0%)	1/2
Ritmeijer *et al.* (2006) [41]	Ethiopia	not reported	RCT	290	**0**	256 (88.3%)	23/6	5/0	157 (54.1%)	30 (10.3%)	60/9

*at 6 months post-treatment, unless indicated otherwise;

# death not directly related to VL;

°criteria for cure not comparable with other trials.

**Table 7:** Pediatric studies.

Study	Country	Year	Studytype	No.	Early treatment Outcome			Late treatment Outcome*		
					Clinicalcure (%)	failure/death	missing/ttm switch	Cure (%)	Relapse (%)	Lost to Follow-up/i.death^#^
Sundar *et al.* (2003) [22]	India	1999–2000	Phase 2	18	18 (100%)	0/0	0/0	15 (83.3%)	2 (11.1%)	1/0
Bhattacharya *et* *al.* (2004) [40]	India	2001–2002	Phase 3	80	79 (98.7%)	0/1	0/0	75 (93.7%)	3 (3.7%)	1/0
Bhattacharya *et* *al.* (2007) [21]	India	2003–2004	Phase 4	411	406 (98.8%)	5		335 (93.6%)	23 (6.4%)	
Singh *et al.* (2006) [42]	India	2003–2005		64	63 (98.4%)	1/0	0/0	59 (92.2%)	1 (1.6%)	3/0

The higher relapse rate in children after miltefosine treatment might also have a pharmacological cause. Miltefosine is only slowly cleared from the human body by phospholipases [23]. Dorlo *et al.* demonstrated that children are significantly less exposed to miltefosine than adults when receiving a similar 2.5 mg/kg/day dosage of miltefosine [24] and proposed a new dosing algorithm to solve this apparent difference in drug exposure between age and body-size groups. Although the exact therapeutic window of miltefosine remains unknown, the first pharmacokinetic-pharmacodynamic relationship for miltefosine in VL has recently been identified. Miltefosine drug exposure (in this case the time that the miltefosine plasma concentration in patients was above 10×EC_50_ in vitro susceptibility of the parasite) was significantly associated with the probability of relapse of disease in patients [25]. Similarly, the link between higher failure rates in children and decreased drug exposure in comparison to adults treated with a similar mg/kg dosage has previously been described as well for other antileishmanial drugs, such as meglumine antimoniate [26], [27].

Prevalence and intensity of infectious diseases are commonly higher in males than in females, as seen with protozoa, nematodes, trematodes, cestodes and arthropods [28]. There are two main hypotheses that explain this observation. The behavioral hypothesis emphasizes gender-related differences in exposure while the physiological hypothesis stresses that genetic and immunological differences may lead to increased susceptibility in males, i.e. linked to circulating steroid hormones [29]. Such male predisposition has been found in the incidence of leishmaniasis, in cutaneous [30] as well as visceral leishmaniasis [31]–[34]. In our cohort, the discrepancy between relapse rates of men and women was present in all age groups and higher in the older (>9 yrs) (table 2, fig. 2), and in all health facilities where relapses were recorded. We found no arguments for behavioral differences with regards to adherence between male and female patients nor between younger and older age groups. In an adherence sub-study in the three participating hospitals in Nepal, adherence defined as 90% of all capsules in the complete treatment regimen taken was 83%, with male sex even being a predictor of good adherence (OR = 2.60, 95% CI 1.02–6.67) [13]. Moreover, almost half of the patients (i.e. those treated at KAMRC) had received miltefosine under DOT.

Differences of statistical significance in treatment outcome for VL between male and female (nor between age groups) have not been reported in clinical trials with other VL drugs, possibly obscured by limited sample sizes, low failure rates and shorter durations of follow-up. Also with pentavalent antimony, where large treatment failure rates (65%) have been recorded, no clinical or biochemical characteristics could be identified that predicted failure [35]. In another cohort in Bihar, unresponsiveness to sodium stibogluconate at the end of treatment was higher in women (48%) compared to men (40%) [36], but this difference was not statistically significant. But in the phase 4 trial with miltefosine [20] the sub-analysis in under-12s shows a failure rate of 8.7% in boys and 3.3% in girls (P = 0.04).

These findings suggest that poorer treatment outcomes i.e. increasing relapse rates under routine program conditions in South-East Asia should not necessarily be seen as an early sign of emerging resistance in the parasite, but can be explained by the enlargement of inclusion criteria to younger age groups with possibly different immune responses against the parasite infection and/or insufficient drug exposure.

More studies are required to help us understand the immunological and pharmacokinetic intricacies that determine success or failure of available and future treatments, both in immunocompetent and HIV co-infected VL patients. Close monitoring of the parasites emerging from the relapses is also recommended, in order to follow the rise of molecular adaptations, like drug resistance or increased virulence, as recently observed in miltefosine relapsing cases [16].

Our findings underscore the need for a close monitoring of treatment outcomes, as well as a critical analysis of the data generated through this monitoring. They highlight the importance of proper clinical trials in children, including pharmacokinetics, to determine the safety, efficacy, drug exposure and therapeutic response of new drugs for neglected diseases, even if running such studies might be extra challenging in the light of ethical regulations. Extrapolation of adult studies is insufficient, and use in infants and adolescents may not necessarily yield comparable results or may even be harmful. In VL, which is a disease affecting often the younger age groups (more so even in East-Africa than in SE Asia), such targeted phase 4 studies and pharmacokinetic studies in children are morally imperative [37].

## Supporting Information

Figure S1 **Flowchart of treatment outcome per clinical setting.**
(TIFF)

Table S1 **Definitions for ITT and PP analysis.**
(DOCX)

Table S2 **Characteristics of included (full follow-up available, at least up to 6 months) and excluded (treatment not completed** ***or*** **lost to follow-up) patient data.**
(DOCX)
