# Supplementary figures and images for: Failure of Miltefosine Treatment for Visceral Leishmaniasis in Children and Men in South-East Asia
Source: PLoS One. 2014 Jun 18;9(6):e100220. doi: 10.1371/journal.pone.0100220 (PMC4062493; doi:10.1371/journal.pone.0100220)

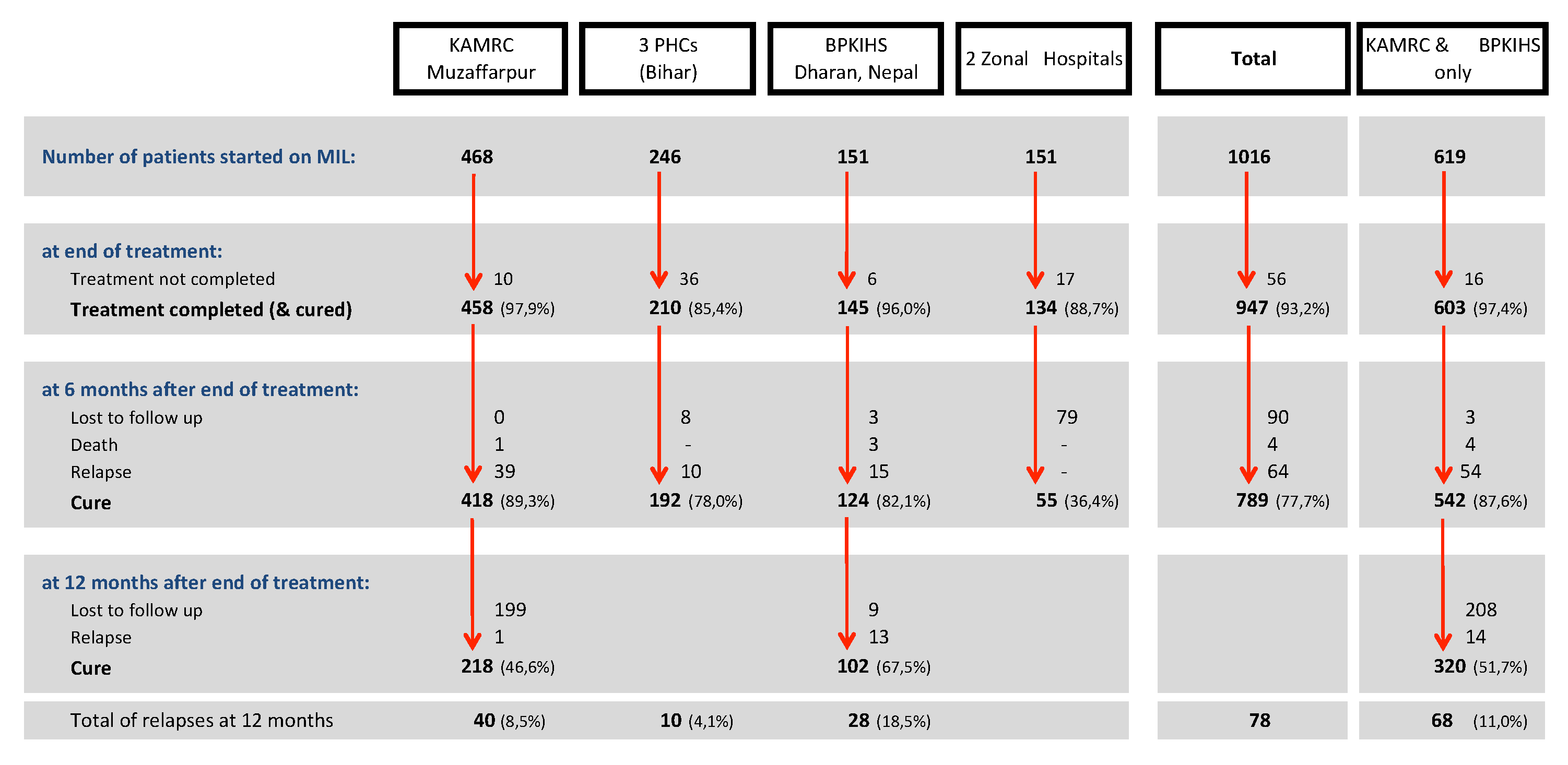

Supplement: Figure S1 — Flowchart of treatment outcome per clinical setting. (TIFF) [file pone.0100220.s001.tiff]
