# Supplementary material for: Failure of Miltefosine Treatment for Visceral Leishmaniasis in Children and Men in South-East Asia
Source: PLoS One. 2014 Jun 18;9(6):e100220. doi: 10.1371/journal.pone.0100220 (PMC4062493; doi:10.1371/journal.pone.0100220)
Supplement: Table S1 — Definitions for ITT and PP analysis. (DOCX) [file pone.0100220.s002.docx]

**Table S1. Methodology: Definitions for ITT and PP analysis**

|  |  |  |  |
| --- | --- | --- | --- |
|  | **Definitions of ITT and PP analysis** | |  |
|  | (non randomised trial, treatment allocation based on treatment guidelines) | |  |
|  |  |  |  |
|  | **ITT (intention to treat)** | |  |
|  |  | including all those who started on MIL treatment and discontinued treatment (default, switch) |  |
|  |  |  |  |
|  |  | exclusion of those lost to follow-up (at the considered time-point) |  |
|  |  | exclusion of those who died for VL-unrelated causes |  |
|  | **ITT worst case** | |  |
|  |  | including all those who started on MIL treatment regardless of treatment protocol completion |  |
|  |  | including those lost to follow-up, whereby these are considered as failures |  |
|  |  | (inclusion of those who died for VL-unrelated causes: they could have relapsed later but they are now "lost to follow up" because of their premature death) | |
|  | **PP (per protocol)** | |  |
|  |  | excluding those who did not complete treatment due to default, severe adverse events of transfer to another health facility (mainly because of complications) | |
|  |  | including those who died under treatment |  |
|  |  | excluding those who died for VL unrelated causes |  |
