# Supplementary material for: Failure of Miltefosine Treatment for Visceral Leishmaniasis in Children and Men in South-East Asia
Source: PLoS One. 2014 Jun 18;9(6):e100220. doi: 10.1371/journal.pone.0100220 (PMC4062493; doi:10.1371/journal.pone.0100220)
Supplement: Table S2 — Characteristics of included (full follow-up available, at least up to 6 months) and excluded (treatment not completed or lost to follow-up) patient data. (DOCX) [file pone.0100220.s003.docx]

**Table S2. Characteristics of included and excluded**

| **Factors** | **Included (853)** | **Treatment not completed (69)** | **Lost to follow-up (94)** | ***P*-value**  (ANOVA) |
| --- | --- | --- | --- | --- |
|  | No. (%) | No. (%) | No. (%) |  |
| Age (year) |  |  |  |  |
| Median (interquartile range) | 20 (11 – 37) | 28 (12 – 40) | 22.5 (11 – 40) | 0.3326 |
|  |  |  |  |  |
| Age groups |  |  |  |  |
| 2-9 | 154 (18.1) | 12 (17.4) | 14 (14.9) |  |
| 10-14 | 177 (20.8) | 9 (13.0) | 21 (22.3) |  |
| 15-24 | 153 (17.9) | 11 (15.9) | 16 (17.0) |  |
| 25 or more | 369 (43.3%) | 37 (53.6) | 43 (45.7) |  |
|  |  |  |  |  |
| Sex |  |  |  |  |
| Male | 525 (61.5%) | 44 (63.8%) | 56 (59.6%) |  |
| Female | 328 (38.5%) | 25 (36.2%) | 38 (40.4%) |  |
|  |  |  |  |  |
| Previous treatment for KA |  |  |  |  |
| No | 745 (87.3%) | 63 (91.3%) | 81 (86.2%) |  |
| Yes | 106 (12.4%) | 5 (7.2%) | 7 (7.4%) |  |
| Missing | 2 (0.2%) | 1 (1.4%) | 6 (6.4%) |  |
|  |  |  |  |  |
| Side effects reported | 172 (20.2%) | 24 (34.8%) | 22 (23.4%) |  |
| …. |  |  |  |  |
